# Early acclimatization to high altitude: Acid–base and fluid balance dynamics during the first 2 days at 3100 m

**DOI:** 10.1113/EP093029

**Published:** 2025-08-25

**Authors:** Elisabeth Skalla, Benedikt Treml, Sasa Rajsic, Michael Schreinlechner, Zoran Bukumirić, Klaus Berek, Alexander Egger, Johann Knotzer, Martin Burtscher, Axel Kleinsasser

**Affiliations:** ^1^ Department of Anaesthesiology Medical University of Innsbruck Innsbruck Austria; ^2^ Department of Radiology Medical University of Innsbruck Innsbruck Austria; ^3^ Department of Cardiology Medical University of Innsbruck Innsbruck Austria; ^4^ Institute of Medical Statistics and Informatics, Faculty of Medicine University of Belgrade Belgrade Serbia; ^5^ Department of Neurology Medical University of Innsbruck Innsbruck Austria; ^6^ Central Institute of Clinical and Chemical Laboratory Diagnostics University Hospital of Innsbruck Innsbruck Austria; ^7^ Department of Anaesthesiology Klinikum Wels Grieskirchen Austria; ^8^ Department of Sports Medicine University of Innsbruck Innsbruck Austria

**Keywords:** acclimatization, bicarbonate, renal reactivity

## Abstract

Immediate responses to hypoxia at high altitude are hyperventilation and successive respiratory alkalosis. Alkalosis, in turn, can affect cerebrospinal fluid pH and ventilatory control. The kidneys compensate metabolically for respiratory alkalosis. The time line and detailed sequence of these renal compensatory processes have not been explored thoroughly. We examined the initial adjustments of acid–base and fluid balances during the first 2 days at high altitude.  Twelve unacclimatized adults of either sex were transported to 3100 m a.s.l. at the Sonnblick Observatory (Austria). Measurements (fluid and acid–base balance and arterial blood gases) were performed before and 24 and 44 h after arrival. Exposure to high altitude provoked hyperventilation, resulting in hypocapnia and alkalosis. Altitude diuresis started immediately after arrival at altitude. The only metabolic response within the first 24 h was a slight reduction in plasma bicarbonate, but after 44 h distinct reductions in bicarbonate and a trend change in altitude‐corrected base excess and arterial pH were observed. Hyperventilation and increased diuresis appeared immediately upon exposure to high altitude, whereas compensatory bicarbonate excretion showed the first influence on the arterial pH at the 44 h measurement. Further research is needed to explore differences in individual responses in the setting of antedated or carry‐over acclimatization.

## Abstract (highlights)

- **What is the central question of this study?**
  Hyperventilation, the immediate response to hypoxia, leads to respiratory alkalosis at high altitude. Renal compensation of altitude‐alkalosis is thought to take days. We studied this during a 44 h sojourn at 3100 m.
- **What is the main finding and its importance?**
  After 24 h at altitude, respiratory alkalosis was compensated only by a subtle reduction in plasma bicarbonate, while base excess and arterial pH remained at the alkalotic end of their normal ranges. After 44 h at altitude, bicarbonate had fallen by 12%, and the base excess and pH had started to normalize. Despite immediacy of the renal response, relevant alkalosis compensation begins with a delay of >24 h.

## 1 INTRODUCTION

Millions of tourists take to the mountains every year (Burtscher et al., 2021). At altitudes of >2000 m, hypobaric hypoxia can cause acute mountain sickness or high‐altitude pulmonary oedema (HAPE). Acute mountain sickness can progress to the potentially fatal high‐altitude cerebral oedema (Hackett and Roach, 2001) and is thus of particular interest. The lack of oxygen plus relative hypoventilation seem to be the driving forces behind these conditions, and redistributions of fluids are central in their development. When the ascent to altitude is passive and rapid, such as in snow sports (by funicular) or as in arriving by aeroplane at, for example, Lukla (Nepal) or Lhasa (Tibet) or La Paz (Bolivia) airports for trekking, altitude exposure is immediate, and an acute response to hypoxia is vital.

### 1.1 Responses to exposure to hypoxia at high altitude

Exposure to hypoxia induces a countless number of physiological adaptions, and the changes in respiration, acid–base balance, and fluid handling are closely coupled upon arrival at high altitude. Hyperventilation causes hypocapnia, alkalosis and a corresponding renal release of bicarbonate, which has a diuretic effect. Bicarbonate diuresis plus sodium and consequent water loss are the backbone of altitude diuresis. In addition, dry air and tachypnoea aid dehydration. The net result is a plasma volume contraction, causing an increased hemoglobin concentration which, together with an increased cardiac output, helps to maintain oxygen delivery to the tissues (Schlitter et al. 2021; Bärtsch & Saltin 2008; Vogel & Harris, 1967).

### 1.2 Metabolic response to hypocapnia and alkalosis

Inadequate altitude diuresis and fluid retention can lead to the development of localized oedema. In a short communication by our group, we compared two women after transport to 3480 m (Kleinsasser et al., 2020). One had no history of HAPE, hyperventilated at altitude, showed plasma volume contraction and preserved the arterial oxygen content well. The other woman, with a history of HAPE, did not increase ventilation, pooled fluids in the plasma and such had less oxygen availability in the arterial blood. In this way, diverse fluid handling affected the amount of oxygen in the arterial blood.

### 1.3 Objectives and hypotheses

Some loose ends remain regarding time course and extent of fluid redistributions, diuresis and acid–base compensations in early acclimatization (Wang et al., 2022), specifically in the course of the first 2 days after arrival at high altitude. This is also the period when most symptoms of acute mountain sickness (and HAPE or high‐altitude cerebral oedema) develop. In this study, we sought to look more closely at these 2 days of primary adaptions and early acclimatization.

The acid–base balance in early acclimatization is closely related to respiration and fluid balance. The kidneys compensate for the respiratory alkalosis secondary to hyperventilation via diuresis with enhanced bicarbonaturia. This process is thought to take days and still to be incomplete thereafter (Steele et al., 2022). This compensation is also considered to be faster at lower altitudes and to show plasticity (Zouboules et al., 2018), but when and in which form the initial response starts has not yet been shown. Given that the carbonic anhydrase reaction itself is simple and fast (Dobyan & Bulger, 1982) and that diuresis is enhanced upon arrival at altitude, we hypothesized that substantial metabolic compensation of alkalosis would be measurable after 24 h rather than after days. There are previous important publications on the issue of early bicarbonate compensation including the work of Gledhill (Gledhill et al., 1975), Dempsey (Dempsey et al., 1975) and Siesjo (Siesjö, 1971). However, simultaneous observations of fluid shifts, weight, pH changes, and base excess upon arrival and the course of the first 2 days at altitude seem still missing. An interesting recent paper displays renal acid–base balance acclimatization after a flight to Las Vegas and a drive to 3800 m within 24 h and a week later (Bird et al., 2021). Nevertheless, changes between the first and second day at altitude are not highlighted.

To address this issue, we looked at this period and included calculation of the extracellular fluid base excess (*ecf* BE). Simultaneously, early responses of the juxtaglomerular apparatus of the kidneys (renin) were monitored.

## 2 MATERIALS AND METHODS

The general concept was to examine volunteers at low altitude, transport them to high altitude and repeat all measurements after 1 and 2 days of exposure to hypobaric hypoxia. No further climbing or exercise was scheduled, and participants had to remain at the same altitude (‘staging’), (Beidleman et al., 2018).

### 2.1 Participant recruitment

Participants were recruited on a voluntary basis, had agreed to repeated blood sampling by individual puncturing of the radial artery and a forearm or cubital vein. We aimed for healthy, active subjects. The main inclusion criterion was thus an age >18 years, and main exclusion criteria were pre‐existent disease, poor altitude tolerance or even a history of altitude disease. The ethics board of the Innsbruck Medical University granted approval (approval number 1111/2022). The experiment was performed in April 2023.

### 2.2 Subjects

Informed written consent was obtained from all 12 participants in this study. This work conforms to the standards set by the *Declaration of Helsinki* (last modified in 2013). The 12 healthy volunteers examined were aged from 30 to 70, with five females and seven males in their third (two), fourth (four), fifth (four), sixth (one) and seventh (one) decade of life. Two of the younger subjects (34 and 41 years of age) were characterized as having low to moderate physical activity, whereas the rest had higher physical activity, including four sports teachers and one mountain guide. The mean age was 50 years, and mean body mass index was 23.0 kg m^−2^. All lived in lowland areas at ∼600 m a.s.l., all were non‐smokers, and none had a recent (within the previous 1 month) exposure to high altitude. In particular, no one had a history of neurological disease.

### 2.3 General measurements

We took all baseline measurements at an altitude of 575 m (University Hospital of Innsbruck, Innsbruck, Austria). Barometric pressure on the day when measurements were taken was 715 Torr. Participants were then transported by car to Kolm Saigurn, Salzburg, Austria, then by using the funicular to the Sonnblick Observatory at 3100 m a.s.l. (www.sonnblick.net/en/). Barometric pressure at the observatory was 511 Torr upon arrival and varied only slightly over the study period. Measurements and blood samples at the observatory were taken after continuous exposure to altitude for 24 and 44 h. Apart from urine composition analyses, all measurements were taken at all three time points. Measurements and calculations are outlined below.

### 2.4 Weight and fluid balance

All subjects were weighed before and at 24 and 44 h after the passive ascent. Urine production was measured and recorded at 24 and 44 h at altitude. Fluid intake was recorded to calculate fluid balance.

### 2.5 Plasma volume

Plasma volume was calculated by the classical approach of Dill and Costill (1974), using hemoglobin and haematocrit levels. Starting with an assumed volume of 100 mL of blood at the lowland measurement, blood and blood plasma volumes at altitude were calculated.

### 2.6 Arterial blood gas analyses

Arterial blood samples were obtained and analyzed immediately using a blood gas analyzer (ABL 80 Flex, Radiometer, Copenhagen). Parameters included the arterial partial pressure of oxygen ($P_{aO_{2}}$), arterial partial pressure of carbon dioxide ($P_{\mathrm{aC}O_{2}}$), arterial pH, sodium, potassium, chloride, calcium and l‐lactate. Other parameters calculated and emitted by the blood gas analyzer were actual bicarbonate (see section 2.8) and the anion gap (see section 2.9).

### 2.7 Extracellular base excess in the lowlands and at altitude

According to Ole Siggaard‐Andersen (1971), the extracellular (fluid) base excess (*ecf* BE) reflects the blood plus interstitial fluid as one buffer compartment. The *ecf* BE is thus of particular interest in the context of metabolic changes of the acid–base status. Zubieta‐Calleja adapted the classic Van Slyke equation for application at altitudes (Van Slyke, 1917; Siggaard‐Andersen & Siggaard‐Andersen, 1995; Zubieta‐Calleja et al., 2011), because otherwise the *ecf* BE resulted in negative values. Accordingly, we used both the sea‐level formula and the formula adapted for an altitude of ≤3500 m by Zubieta‐Calleja et al. (2011):

$$\begin{array}{ccc} Ecf\mathrm{BE}\;\mathrm{for}\;\mathrm{the}\;\mathrm{lowland}\;\mathrm{measurement} & = & 0.93\times \left[\Delta (\mathrm{HC}{O_{3}}^{-})\right.\\ & & \left.+\;14.6\times \Delta \mathrm{pH}\right], \end{array}$$

where Δ (HCO_3_^−^) = actual bicarbonate − 24.5.

$$\begin{array}{ccc} \;Ecf\mathrm{BE}\;\mathrm{for}\;\mathrm{the}\;\mathrm{measurement}\;\mathrm{at}\;3100\;m & = & 0.92\times \left[\Delta (\mathrm{HC}{O_{3}}^{-})\right.\\ & & \left.+\;15.1\times \Delta \mathrm{pH}\right], \end{array}$$

where Δ(HCO_3_^−^) = actual bicarbonate − 18.0.

Please note that *ecf* BE was eventually described as (negative) titratable hydrogen ion concentration according to Ole Siggaard‐Andersen (2004). Titratable hydrogen ion concentration equals *ecf* BE, but with opposite sign; however, we use the term *ecf* BE in this work because this is the more familiar name for most readers.

### 2.8 Actual bicarbonate

In all further calculations, we used ‘actual’ bicarbonate, calculated from arterial pH and $P_{\mathrm{aC}O_{2}}$ using the classic Henderson–Hasselbalch equation. These calculations were performed by the algorithm of the blood‐gas analyzer used (ABL 80 Flex, Radiometer, Copenhagen).

### 2.9 Anion ion gap

The anion ion gap, reflecting unmeasured anions, was calculated using sodium, potassium, bicarbonate, and chloride.

### 2.10 Calculations of renal reactivity for day 1 and day 2

Calculations of renal reactivity (RR) were performed using:


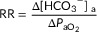




The RR is a recently presented index (Zouboules et al., 2018) that measures relative changes in arterial bicarbonate (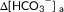) against relative changes in the $P_{\mathrm{aC}O_{2}}$ ($\Delta P_{\mathrm{aC}O_{2}}$) in order to reflect how the kidneys remove bicarbonate in the presence of hypocarbia and alkalosis at altitude.

### 2.11 Renin and cortisol

Renin was measured in blood plasma. Participants rested for 30 min beforehand. Samples were obtained venously, centrifuged, and plasma was stored at 7°C–9°C. Also, blood samples for cortisol were obtained, centrifuged (1500 x g for 15 minutes), and plasma supernatant was frozen using dry ice. Renin and cortisol samples were obtained in the early afternoon. Samples were analyzed using a chemiluminescent assay (IDS‐ivy's direct renin, Immunodiagnostic Systems Limited, Boldon Colliery, UK) and an electrochemiluminescence assay (Elecsys Cortisol II applied on Cobas 8000 analyzer, Roche Diagnostics, Mannheim, Germany).

### 2.12 Urine composition

Urine composition was determined from the samples taken before ascent and after 44 h at altitude. Parameters observed included osmolality, sodium, potassium and chloride. Osmolality was determined using a Fiske 2400 Osmometer) (Fieske Associates, Norwood, MA, USA), and the electrolytes were determined by indirect potentiometry on a Cobas 8000 system (Roche Diagnostics, Mannhein, Germany).

### 2.13 Ventilatory measurements

Minute ventilation (${\dot{V}}_{E}$) was measured before and 24 and 44 h after ascent to high altitude by use of the Spiropalm 6MWT Spirometer (with turbine flowmeter; Cosmed, Italy) on a breath‐to‐breath basis, SpO_2_ was recorded using a Pulsox‐3i (Minolta, Osaka, Japan), as described in the previous work of our group (Wille et al., 2012).

### 2.14 Circulatory measurements

Circulatory measurements included heart rate and blood pressure and were performed at rest using a non‐invasive monitor (CNAP, CNSystems, Graz, Austria), which delivers accurate measurements (Dewhirst et al., 2013).

### 2.15 Altitude sickness

Questionnaires for altitude‐associated sickness were answered at 575 m and after 24 and 44 h at 3100 m. In this trial, we used the 2018 edition of the Lake Louise Score (Roach et al., 2018) and the Acute Mountain Sickness Cerebral score, which is derived from the Environmental Symptom Questionnaire (ESQ‐III) (Bärtsch et al., 2004).

### 2.16 Statistical analyses and graphics software

**FIGURE 1:**
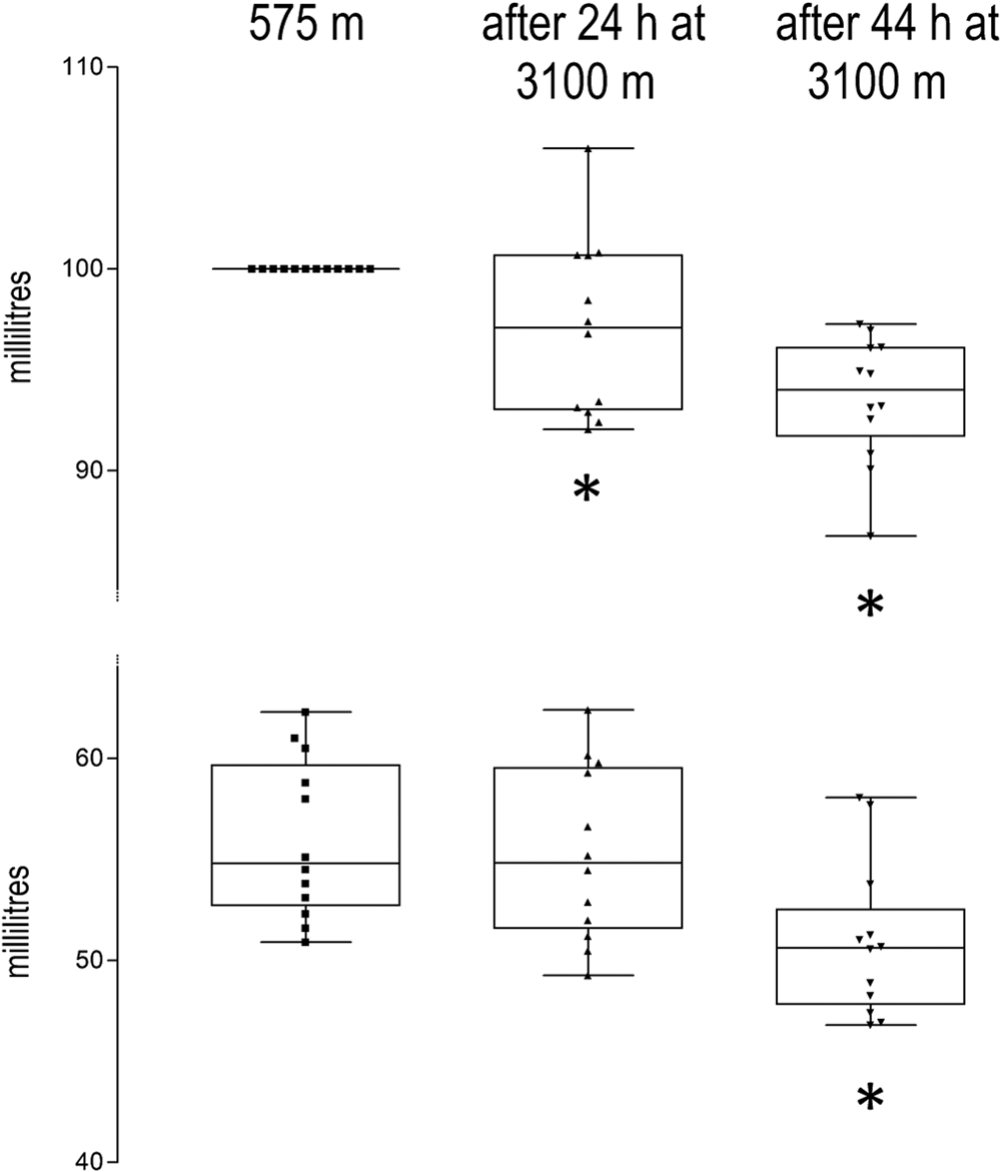
Plasma (a) and cell volume (b) at 575 m (lowland time point) and after 24 and 44 h at 3100 m. (a) Volumes are calculated using the classic formula of Dill and Costill (1974) in reference to the lowland values. Asterisk (^*^) denotes significant (*P *= 0.011) change in plasma volume. Cell volume did not change.

**FIGURE 2:**
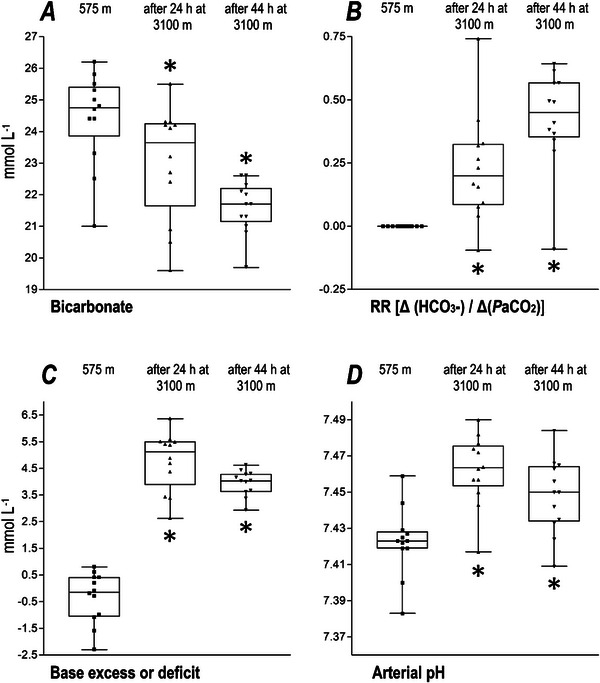
Renal reactivity and acid–base balance in early acclimatization. (a) Decrease in bicarbonate over time. After 24 h at 3100 m, bicarbonate had fallen (*P *= 0.048), and more so after 44 h, where the probability of error was *P *< 0.001 in comparison to the lowland measurement at 575 m. (b) Renal reactivity in the lowlands is zero and rises after 24 h at 3100 m (*P *= 0.004) and after 44 h (*P *= 0.031 vs. the 24 h measurement). (c) Altitude‐corrected base excess in the extracellular fluid increases steeply after 24 h (*P *< 0.0001), then decreases as recorded at the 44 h measurement (*P *= 0.0032 vs. the 24 h measurement). (d) Arterial pH increases in the first 24 h at altitude as a consequence of the lowered arterial partial pressure of carbon dioxide (*P *< 0.01), then decreases (*P *= 0.005) versus the 24 h measurement with metabolic compensation [i.e., with the bicarbonate loss displayed in (a), dynamism expressed as RR in (b)]. ^*^Significant difference. Abbreviation: RR, renal reactivity.

Statistical analyses were performed by using SPSS (v.22.0, released 2013, IBM Corp., Armonk, NY, USA) Missing data were not analyzed. Depending on the normality of the distribution and the type of variable, results are presented as frequency (%), mean ± SD. For parametric data, a paired‐samples, repeated‐measures *t*‐test was used. In this manner, we compared all measurements taken at altitude with renal reactivity and arterial pH was assessed using the Spearman Correlation coefficient. Plotting was done using GraphPad Prism v.3.0 (GraphPad, San Diego, CA, USA) and GIMP (Gnu Image Manipulation Program v.2.10.38) was used for labelling graphs. Classic box‐and‐whisker plots were used for Figures 1 and 2, where the box is made up from the upper and lower quartiles and a horizontal bar denoting the median. Upper and lower extremes are shown as the whiskers.

## 3 RESULTS

**FIGURE 3:**
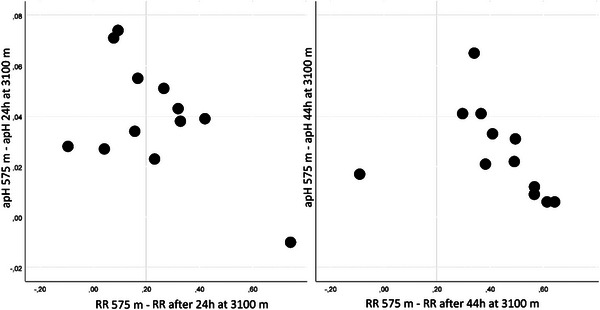
Renal reactivity plotted against arterial pH. This figure shows that the increase in RR is positively correlated with a reduction in arterial pH between the 24 and 44 h measurements (right panel, *P *= 0.007) but not in the first 24 h at 3100 m (left panel, *P *= 0.602). Abbreviations: apH, arterial pH; RR, renal reactivity.

Results are presented narratively in this section and shown in Figures 1, 2, 3 and in Tables 1 and 2; *n* is 12 throughout.

**TABLE 1:** Changes in arterial blood variables before and 24 and 44 h after passive ascent to 3100 m.

Variable (mean ± SD)	575 m (*n* = 12)	After 24 h at 3100 m (*n* = 12)	After 44 h at 3100 m (*n* = 12)
$P_{aO_{2}}$, mmHg	84.44 ± 9.75	59.52 ± 4.21^*^	58.81 ± 3.24^*^
$S_{aO_{2}}$, %	96.48 ± 1.71	91.53 ± 2.02^*^	91.58 ± 1.87^*^
$P_{\mathrm{aC}O_{2}}$, mmHg	37.46 ± 2.99	32.19 ± 2.48^*^	31.27 ± 1.80^*^
Na^+^, mmol L^−1^	141.4 ± 1.5	142.4 ± 1.7	141.4 ± 1.4
K^+^, mmol L^−1^	4.06 ± 0.23	3.86 ± 0.20	3.98 ± 0.30
Cl^−^, mmol L^−1^	107.42 ± 1.51	109.25 ± 1.77^*^	108.92 ± 1.08^*^
Renin, ng L^−1^	16.50 ± 8.92	7.35 ± 3.18^*^	7.66 ± 3.17^*^
Cortisol, µg dL^−1^	101.71 ± 29.08	88.44 ± 18.23	144.02 ± 37.68^*^
Anion gap, mequiv L^−1^	13.76 ± 1.125	14.10 ± 1.249	14.86 ± 1.026^*^
Haematocrit	44.01 ± 3.96	43.02 ± 3.75	45.60 ± 3.61

Abbreviations: $P_{\mathrm{aC}O_{2}}$, arterial partial pressure of carbon dioxide; $P_{aO_{2}}$, arterial partial pressure of oxygen; $S_{aO_{2}}$, oxyhaemoglobin saturation.

* *P* < 0.05 in comparison to baseline (575 m) value.

**TABLE 2:** Mean changes in cardiac and circulatory variables.

Variable (mean ± SD)	575 m (*n* = 12)	After 24 h at 3100 m (*n* = 12)	After 44 h at 3100 m (*n* = 12)
Systolic BP, mmHg	123 ± 14	118 ± 8	126 ± 17
Diastolic BP, mmHg	76 ± 8	76 ± 6	78 ± 10
Mean arterial BP, mmHg	89 ± 10	88 ± 87	92 ± 11
Heart rate, beats min^−1^	65 ± 6	77 ± 7^*^	78 ± 9^*^

Abbreviation: BP, blood pressure.

* *P *< 0.05.

### 3.1 Weight and fluid balance

Participants lost 436 g (mean) of body weight during the observation period. Urine output was 2464 mL in the first 24 h and 3388 mL in the second 20 h. Mean urine production was 1.4 mL kg^−1^ h^−1^ in the first 24 h and 2.3 mL kg^−1^ h^−1^ in the second 20 h. The resulting fluid balance was −819 mL in the first 24 h and +17 mL in the second 20 h, resulting in a net fluid balance of −644 mL over 44 h at altitude.

### 3.2 Plasma and cell volume

Plasma and cell volume are displayed in Figure 1. Plasma volume was contracted at the 44 h time point (*P *= 0.011), which is a 7% contraction in comparison to the lowland value. Cell volume did not change over the 44 h observation period.

### 3.3 Arterial blood gas analyses

The three parameters traditionally considered for arterial blood gas analysis ($P_{aO_{2}}$, $P_{\mathrm{aC}O_{2}}$ and arterial pH) changed in the known and expected manner, whereby $P_{aO_{2}}$ decreased and $P_{\mathrm{aC}O_{2}}$ decreased, resulting in an elevated arterial pH at the 24 h measurement (Table 1; Figure 2). At the 44 h measurement, arterial pH had started to fall (Figure 2), while *P*aCO_2_ remained unchanged in comparison to the 24 h measurement.

### 3.4 Extracellular fluid base excess in the lowlands and at altitude

The *Ecf* BE (corrected for altitude) increased during the first 24 h at altitude (*P *< 0.001) but had begun to descend at the 44 h measurement in comparison to the lowland measurement (*P *= 0.001; Figure 2).

### 3.5 Bicarbonate, chloride, anion gap, and l‐lactate

Bicarbonate in the blood plasma was lower at the 24 and 44 h measurements in comparison to the lowland level. Bicarbonate values are displayed in Figure 2. Chloride, the second major anion in the plasma, was not measurably retained by the kidneys (in exchange for bicarbonate excretion) in the presence of l‐lactate increases from (mean ± SD) 5.8  ±  2.2 mg dL^−1^ (lowlands) to 13.1  ±  7.6 mg dL^−1^ (after 24 h at 3100 m, *P *= 0.004) and 15.8  ±  6.3 mg dL^−1^ (after 44 h at 3100 m, *P *< 0.001). Accordingly, the anion gap showed only a trend towards an increase (Table 1).

### 3.6 Renal reactivity

The RR is presented in Figure 2. The RR is calculated versus lowland values of bicarbonate and $P_{\mathrm{aC}O_{2}}$, hence there is no real ‘lowland RR’. Setting the lowland value to zero and comparing the value at 24 h at altitude with this virtual lowland RR (i.e., zero) resulted in *P *= 0.004. The 44 h at altitude comparison versus lowland resulted in a *P* = 0.001.

The change in RR was also plotted and correlated against the change in arterial pH. Here, we found a positive inverse correlation (*P *= 0.007) between the increase in RR from 24 to 44 h at altitude and a corresponding reduction in arterial pH in the same period (Figure 3). For the first day at altitude, this was not the case (*P *= 0.602; Figure 3).

### 3.7 Renin and cortisol

Renin was reduced at the 24 and 44 h measurements in comparison to baseline, whereas cortisol was elevated only at the second time point at altitude (Table 1).

### 3.8 Urine composition

The urine samples obtained before ascending the mountain contained 112 ± 48 mmol L^−1^ sodium, 115 ± 57 mmol L^−1^ chloride and 48 ± 31 mmol L^−1^ potassium, and osmolarity was at 572 mosmol L^−1^. After 44 h at altitude sodium, chloride and potassium were measured at 109 ± 37 (*P = *0.88), 122 ± 52 (*P *< 0.001) and 47 ± 32 mmol L^−1^ (*P *= 0.002), respectively, while osmolality had changed to 530 ± 213 mosmol L^−1^ (*P *= 0.67). Although some differences were statistically significant, the changes in absolute terms were small.

### 3.9 Ventilatory measurements

Resting ${\dot{V}}_{E}$ was 7.70 ± 0.92 L min^−1^ at low altitude, 10.20 ± 2.48 L min^−1^ after 24 h at high altitude and 10.4 ± 2.07 L min^−1^ after 44 h at high altitude.

### 3.10 Circulatory measurements

Circulatory measurements revealed well‐known changes that occur upon exposure to hypoxia at altitude. Values are displayed in Table 2.

### 3.11 Altitude sickness

None of the participants developed acute mountain sickness during the stay at high altitude. The Lake Louise Score was 0.42 ± 0.67 after 24 h at high altitude and 0.08 ± 0.29 after 44 h.

## 4 DISCUSSION

In this field study on the initial acclimatization response to sudden exposure to hypoxia/high altitude, we looked at acid–base compensation by the kidneys and at fluid balance during the first 44 h at hypobaric hypoxia. The kidneys counteract respiratory alkalosis by the excretion of bicarbonate. Plasma bicarbonate in our subjects had fallen after 24 h. By 44 h at altitude, bicarbonate had fallen again, to 21.6 mmol L^−1^. In terms of RR (ΔHCO_3_^−^/$\Delta P_{\mathrm{aC}O_{2}}$), the kidneys reacted instantly to acute respiratory alkalosis. However, we also noted that the effect of bicarbonate elimination on arterial pH started only on the second day at altitude. This was also true for the altitude‐corrected *Ecf* BE, which was increased at 24 h but had begun to descend at the 44 h measurement (Figure 2). Renal compensation was accompanied by substantial diuresis; however, at 44 h the total fluid balance was only −644 mL.

### 4.1 Early plasma volume contraction: Shift or loss?

Plasma volume contraction has traditionally been attributed to a reduction in total body water as a consequence of altitude diuresis (Siebenmann et al., 2017). However, looking at the more recent literature, a shift in fluid rather than loss of total body water appears to be the prevailing reason in most hard‐to‐control experimental settings (Siebenmann et al., 2024). In our experiment, a 7% plasma volume contraction was observed after 44 h (*P =* 0.011) (Figure 1). In our experiment, the initiation of plasma volume contraction as a relevant acclimatization process began with a delay of >24 h.

We also recorded a slightly negative fluid balance after 44 h at altitude (−644 mL, *n* = 11) and a mean weight loss of 436 g (*n* = 11) in the volunteers observed. Taken together, these findings support the notion that in our experiment, during early acclimatization diuresis outweighs fluid shifts in the generation of altitude‐associated plasma volume contraction.

### 4.2 Renal reactivity in early acclimatization

Renal reactivity is a new and useful parameter indexing arterial bicarbonate changes against changes in $P_{\mathrm{aC}O_{2}}$ (Zouboules et al., 2018). Zouboules and coworkers examined hikers at incremental altitudes in the Everest region. They found that from the first altitude measurements on the third day, renal compensation increases to plateau on the fifth day of incremental ascent to 3820 m a.s.l., indicating plasticity. In our experiment, we shed some light on the first 2 days at high altitude. We found that RR started (from a low‐altitude baseline of zero) at 0.229 after 24 h and increased to 0.423 at the 44 h measurement. This also supports the notion of plasticity of RR. The maximum deflection observed in the experiment by Zouboules et al. (2018) was 0.614, such that about one‐third of the response was reached on the first day and some two‐thirds on the second day in our experiment. Accordingly, plasma bicarbonate in our experiment decreased to the same extent as in the experiment by Zouboules et al. (2018). Renal reactivity is an immediate response, which in its early stages is also apparently plastic.

### 4.3 The information our experiment can add to the experiment by Zouboules and colleagues

The interesting concept of RR at altitude (Zouboules et al., 2018) examines how the kidneys deal with the relative excess of bicarbonate in the presence of hyperventilation with hypocapnia at altitude. In the original experiment, volunteers were examined from the third day at altitude (at Namche, Nepal, 3400 m) onwards, while the participants were constantly ascending. Measurements were taken every second or third day.

In our experiment, we aimed to look at the period beforehand, i.e., the first and second day at altitude, with participants remaining at the same altitude (‘staging’), without physical activity. We think this initial period of staging at altitude is particularly interesting because it has been shown to lower the incidence of mountain sickness when climbing from 3000 m to higher altitudes (Beidleman et al., 2018).

### 4.4 Immediacy of RR: Looking back at a classic publication

We did not take blood immediately upon arrival at altitude, but this has been done in a classic work by Jerome Dempsey in 1974 (Dempsey et al., 1974). Calculating RR using these numbers, we cannot detect an RR at 1 h post arrival (RR = −0.249; *n* = 7), but at 8 h after arrival at altitude the RR index was of similar magnitude to the experiment by Zouboules et al. (2018) and our experiment (RR = 0.309, *n* = 5).

### 4.5 Time to compensation in normobaric and hypobaric hypoxia

How long would it take until metabolic compensation is established? In a recent study on exercise impairment, 16 participants were exposed to normobaric hypoxia with 15% oxygen, simulating 3000 m of altitude (Limmer et al., 2020). In that study, a significant reduction in bicarbonate levels was observed no earlier than after 12 h in a normobaric hypoxic chamber when resting. The difference between the starting level and the first significantly lower level was −1.35 mmol L^−1^. This magnitude is comparable to our values after 24 h (−1.42 mmol L^−1^) and after 44 h (−2.82 mmol L^−1^), with a mean hourly reduction of 0.064 mmol L^−1^. This, in turn, means that a bicarbonate level of 18.3 mmol L^−1^, which is the level in acclimatized individuals at 3100 m (Ramirez‐Sandoval et al., 2016), would be reached in some 95 h (i.e., ∼4 days), given that bicarbonate elimination is a linear function. This might not be the case, taking into account the findings of Zouboules et al. (2018), who found plasticity in RR in hypobaric hypoxia. Clearly, a study in hypobaric hypoxia with serial measurements of $P_{\mathrm{aC}O_{2}}$, arterial pH and bicarbonate over several days is needed to define bicarbonate excretion kinetics better.

### 4.6 Time course of metabolic compensation: Arterial pH and *ecf* BE

The acid–base balance showed only subtle shifts in the first 44 h at altitude. After an initial alkaline deflection at the 24 h measurement, the initial compensatory shift of the arterial pH was observed at the 44 h measurement. Also, *ecf* BE was initially deflected towards the alkaline part of the spectrum, but showed a reverse movement at the 44 h measurement. Only plasma bicarbonate showed an initial downward movement at the 24 h measurement. Complete metabolic compensation of alkalosis will need >44 h, as in our experiment, or might not occur even after 4 or 8 weeks at altitude, given the arterial pH data provided by Dempsey et al. (1974) and Lundby et al. (2004).

### 4.7 Initiation of metabolic compensation: RR and arterial pH

When does metabolic compensation begin? We looked for the moment when RR starts to be related to arterial pH, as demonstrated in the work of Zouboules et al. (2018). Plotting the change in RR against the change in pH, we found an inverse correlation (*P *= 0.007, *r* = −0.733) between the increase in RR from 24 to 44 h at altitude and the corresponding reduction in arterial pH in the same period. However, there was no correlation between RR and arterial pH during the first 24 h at altitude (*P *= 0.602, *r* = −0.168; Figure 3). This is compatible with the notion that the effect of bicarbonate elimination on arterial pH starts on the second day at altitude.

### 4.8 Renin, urine production, and sodium

Renin, a product of the juxtaglomerular apparatus of the kidneys, was reduced in the measurements taken after 24 and 44 h at altitude (Table 1); an immediate response. Our results are in line with a classic publication (Olsen, 1995). Via the renin–angiotensin–aldosterone system and suppression of vasopressin, a decrease in renin helps to increase diuresis. In the present experiment, urine production was 2464 mL in the first 24 h and increased to 3388 mL during the second 20 h at altitude. Renin reduces the retention of sodium; however, sodium levels in the blood plasma did not fall, which might have been associated with no particular restriction in terms of dietary salt. That being said, looking at cortisol which helps to conserve sodium (collecting tubule of the kidneys), we recorded an increase at the 44 h measurement (*P *< 0.05; Table 1).

### 4.9 Critique of methods

Looking back, we regret that we did not perform serial urine analyses on pH. This would have provided insight into the kinetics of bicarbonate excretion. In addition, we did not control nutritional salt intake in this field experiment, which might have yielded more homogeneous results. Moreover, 2 days more at altitude with the same set of measurements every day might have elucidated the total course of RR in healthy adults and added further to the work of Zouboules et al. (2018). In addition, the Dill‐Costill formula (Dill & Costill, 1974) was rather designed for a single before–after comparison than for a serial observation of plasma and cell volumes. In this manner, altitude time points were individually compared to the lowland values.

### 4.10 Future research

Future research should be directed towards the application of pre‐acclimatization, as in a previous experiment by our group (Treml et al., 2020). In this way, one would be able to determine whether pre‐ or carry‐over acclimatization can help to reach a plateau in RR sooner or even raise this plateau.

## 5 CONCLUSION

Bicarbonate excretion is an instant response to the respiratory alkalosis resulting from hyperventilation at altitude. The RR, a parameter of renal compensation of alkalosis, reacts promptly to exposure to high altitude. In arterial pH, *ecf* BE and in plasma volume contraction, however, compensations can be seen beginning on the second day at altitude.

## AUTHOR CONTRIBUTIONS

Elisabeth Skalla and Benedikt Treml contributed equally to this work. Elisabeth Skalla, Axel Kleinsasser, Michael Schreinlechner, Sasa Rajsic, Benedikt Treml, and Alexander Egger. conceptualized the study. Benedikt Treml was responsible for assistance with ethics and organization of the experiment. Elisabeth Skalla organized all aspects of sonographic examinations together with Michael Schreinlechner, Sasa Rajsic took care of all data recording and processing together with Zoran Bukumirić and Klaus Berek. Alexander Egger created ways to store, transport and appropriately analyze data at high altitude. Martin Burtscher performed a great part of the lowland examinations together with Michael Schreinlechner. Johann Knotzer organized workflow at altitude and assured data quality. Elisabeth Skalla and Michael Schreinlechner took care of data interpretation. Elisabeth Skalla, Axel Kleinsasser, Johann Knotzer, Benedikt Treml, and Michael Schreinlechner were responsible for manuscript editing. All authors approved the final version of the manuscript and agree to be accountable for all aspects of the work in ensuring that questions related to the accuracy or integrity of any part of the work are appropriately investigated and resolved. All persons designated as authors qualify for authorship, and all those who qualify for authorship are listed.

## CONFLICT OF INTEREST

The authors declare no conflicts of interest.

## Data Availability

All data supporting the results presented in the manuscript are included in the manuscript figures.
